# Document vectorization method using network information of words

**DOI:** 10.1371/journal.pone.0219389

**Published:** 2019-07-18

**Authors:** Sang Yup Lee

**Affiliations:** Department of Communication, Yonsei University, Seodaemun-gu, Seoul, South Korea; University of Sao Paulo, BRAZIL

## Abstract

We propose a new method for vectorizing a document using the relational characteristics of the words in the document. For the relational characteristics, we use two types of relational information of a word: 1) the centrality measures of the word and 2) the number of times that the word is used with other words in the document. We propose these methods mainly because information regarding the relations of a word to other words in the document are likely to better represent the unique characteristics of the document than the frequency-based methods (e.g., term frequency and term frequency–inverse document frequency). In experiments using a corpus consisting of 14 documents pertaining to four different topics, the results of clustering analysis using cosine similarities between vectors of relational information for words were comparable to (and more accurate than in some cases) those obtained using vectors of frequency-based methods. The clustering analysis using vectors of tie weights between words yielded the most accurate result. Although the results obtained for the small dataset used in this study can hardly be generalized, they suggest that at least in some cases, vectorization of a document using the relational characteristics of the words can provide more accurate results than the frequency-based vectors.

## Introduction

We propose a document vectorization method using the relational characteristics of words in the document. In general, a document is represented as a vector using the bag-of-words model [1]. The entries of the vector are the frequencies of each word in the document (i.e., the term frequency (TF)) [2]. In addition to the TF, the TF–inverse document frequency (TFIDF) is frequently used to better represent the unique characteristics of a document [3].

The main purpose of the vectorization of a document with words is to represent the unique characteristics of the document numerically so that computers can handle such unstructured text data [4]. In many machine-learning algorithms used for text mining, how well the unique characteristics of a document are represented by its words plays a critical role in analyzing the document [5]. For example, when calculating the similarity between two documents, we typically use vectors made of the information about the frequencies of the words used in the document, such as the TF or TFIDF. The accuracy of the similarity is highly dependent on the degree to which the vectors correctly represent the unique characteristics of the documents [6].

However, traditional methods such as the TF and TFIDF have limitations in representing the unique characteristics of a document, mainly because they do not consider important characteristics of the words used in a document, for example, the relations of each word with other words in the document.

To alleviate these limitations, we suggest a new method of document vectorization using the relational characteristics of each word, that is, information about what other words the word is used together with and the frequency of co-occurrence between the word and other words in the document. How (and how often) a word is used with other words in a document can reflect unique characteristics of the document. The author of a document attempts to convey his/her thoughts and opinions using words, not in a random way but in a systematic way. Thus, the topic or content of a document significantly depends on which words are used in the same sentences. For example, even if two different words, e.g., Words 1 and 2 are used with the same frequency (e.g., 10 times) in a document, the case where they are used in the same sentences 10 times differs significantly from the case where they are used in different sentences. In the former case, Word 1 has a close relation to Word 2, whereas in the latter case, the two words have a less close relation. This indicates that if we consider the relational characteristics of words used in a document when vectorizing the document, we can extract the unique characteristics of the document more effectively.

In this study, we attempt to vectorize a document using two types of relational information of a word: 1) the centrality measures of the word and 2) the number of times that the word is used with other words in the same sentences in the document. After introducing the proposed methods, we evaluate their performance by applying them for clustering analysis and compare the clustering results with those obtained via traditional methods, i.e., TF and TFIDF. The main purpose of clustering analysis is to find clusters among different data points, which are documents in this case, based on the similarity between documents. If the vectors represent the unique characteristics of a document better, then it is expected that the similarity (and difference) between documents will be more cleared distinguished, which will lead to more accurate results of clustering analysis.

For the evaluation, we used small text data composed of 14 news articles, each of which covers a particular topic. There are four different topics covered by the 14 articles; thus, the number of clusters that we wish to find is four. We used this small corpus mainly because 1) the documents can be manually labeled according to their topics and 2) we can specifically calculate the similarity between two particular documents and examine where the differences exist between them, which helps compare the new method with the existing methods more clearly.

We found that the clustering analyses based on cosine similarities between vectors of the new methods outperformed those based on the vectors of traditional methods. However, the results of clustering analysis based on the Euclidean distances between document vectors obtained using the proposed methods were less accurate than those resulted from vectors of traditional methods.

Prior studies have shown that network-based approaches can be useful for natural language processing (LNP) tasks [7, 8]. Syntactic and stylistic characteristics of a language can be represented by network-based approaches, thus, they are useful to identify different languages [8, 9]. Networks of words have also been proven to reflect the unique writing style of an author [10]. Word adjacency models have also been corroborated to reflect characteristics of texts [11–15]. In a word adjacency model, a tie is defined to exist between two adjacent words and a document is represented by an adjacency matrix of words [16]. Similarly, the Doc2Vec proposed by Le and Mikolov in 2014 [17], which is based on neural networks, uses information about a word and its context words to represent a document with a low dimensional vector.

## Methods

### Bag-of-words model

We explain the bag-of-words model using the following simple corpus composed of four different documents.

Doc1 = “banana apple apple eggplant”
Doc2 = “orange carrot banana eggplant”
Doc3 = “apple carrot banana banana”
Doc4 = “orange banana grape”

In the corpus, there are six words: “apple,” “banana,” “carrot,” “eggplant,” “grape,” and “orange.” First, we can vectorize each document using the frequency of each word in the corpus. For example, the vector for Doc1 is (2, 1, 0, 1, 0, 0), which indicates that “apple” is used twice in Doc 1, “banana” and “eggplant” are used once, and the other words are not used in Doc 1. By performing similar calculations, we can obtain the document–term matrix shown in Table 1, where each row represents the vector of a document.

**Table 1:** Document–term matrix for the example four documents.

	apple	banana	carrot	eggplant	grape	orange
**Doc1**	2	1	0	1	0	0
**Doc2**	0	1	1	1	0	1
**Doc3**	1	2	1	0	0	0
**Doc4**	0	1	0	0	1	1

To determine the unique characteristics of a document compared with other documents, the TFIDF metric is more frequently used than the TF. The TFIDF of a word in a document is calculated as the product of the TF and IDF. The TF is the number of times that the word is used in the document, whereas the IDF refers to the number of other documents in the corpus that the word is not used [18]. The IDF is calculated as

$$idf(t,D)=\log\frac{\left|D\right|}{\left|(d\in D:t\in d)\right|},$$

where |*D*| is the number of documents in the corpus, and |(*d*∈*D*:*t*∈*d*)| is the number of documents in which the term *t* is used [18]. The IDF has a large value if the term *t* is used in few other documents in the corpus. The TFIDF of a term reflects the relative importance of the term in the document. Terms with a large TFIDF are those that are frequently used in the document but not in other document in the corpus. Thus, terms with a large TFIDF are likely to represent the unique characteristics of the document better than terms with a large TF value [18].

As shown above, TF and the TFIDF are based on the frequency of each word but not other information about a word. Although frequency information can represent the unique characteristics of a document well, sometimes, relational characteristics of words can be more useful.

### Relational characteristics of words in a document

To identify the relational characteristics of the words in a document, that is, how a word is used in relation to other words in the document, we can represent the document as a network composed of the words used in the document. A tie between two words in the network can be defined in several different ways. In this study, a tie exists between two words when they are used in the same sentence. For example, for a sentence “An apple is a sweet, edible fruit produced by an apple tree,” a tie between “apple” and “fruit” is said to exist because they are used in the same sentence. Further, a tie can have particular attribute information called a weight, which refers to the number of times that the two words are used in same sentences. For example, consider the following document, which consists of five sentences.

DocA = *“banana apple carrot. banana grape apple watermelon orange. grape eggplant spinach orange. banana eggplant apple. cherry melon pear eggplant.”*

**Fig 1:**
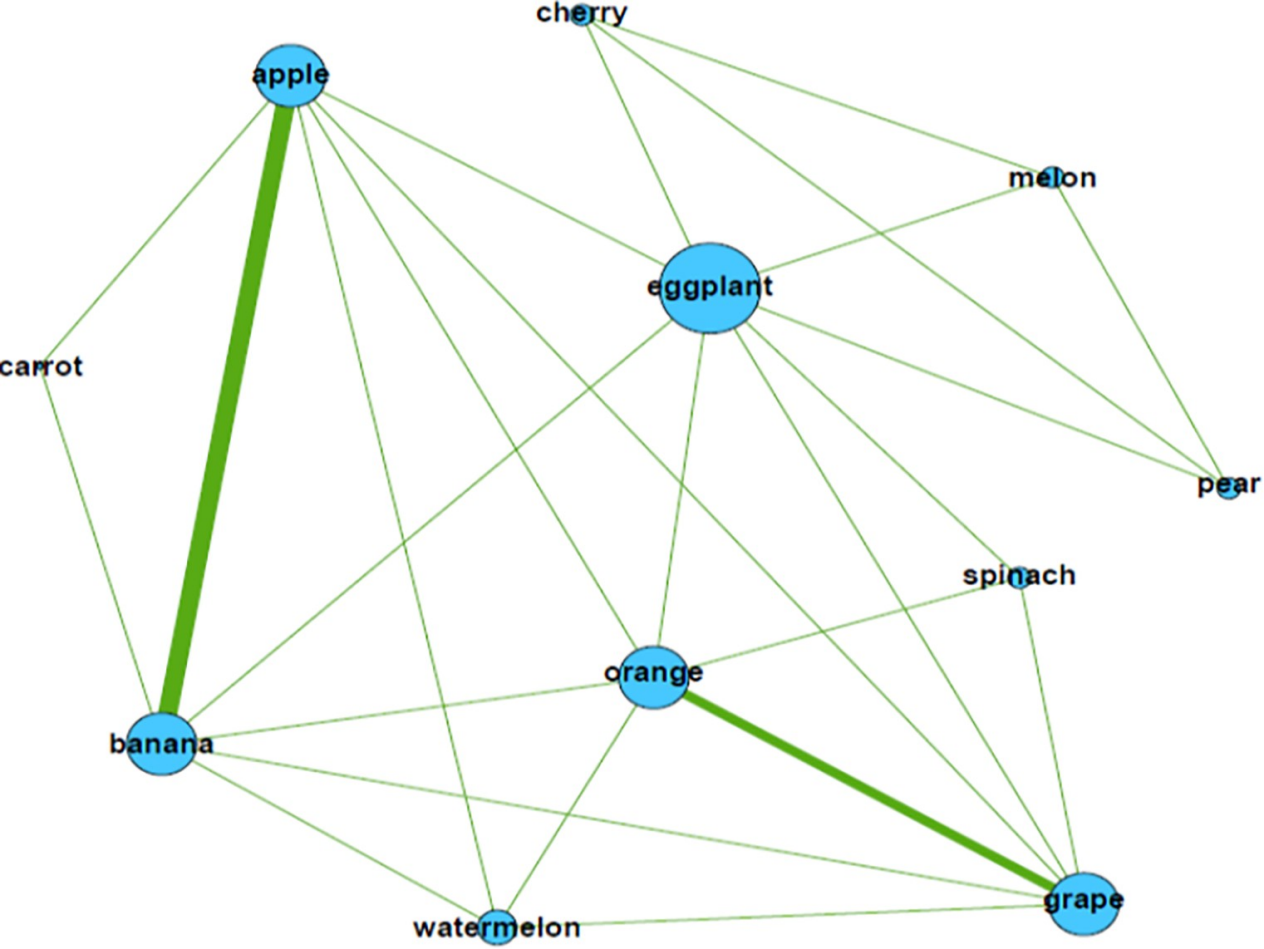
Word network of DocA. * The size of a node refers to the degree of the node, whereas the width of a tie between two nodes refers to the weight of the tie.

The network of words in the document is shown in Fig 1.

The size of each word in Fig 1 indicates the number of adjacent words, which is known as the degree of the node. The adjacent words of a word are the words that are used with the word in the same sentences. As mentioned previously, a tie exists between two words when they are used in the same sentence. For example, there is a tie between “eggplant” and “spinach” because they are used in the third sentence of DocA. The width of a tie reflects the weight of the tie, i.e., the number of times that the two words are used in same sentences. Thus, a thicker tie indicates a larger weight. For example, in DocA, the two words “apple” and “banana” are used more frequently in same sentences than other words. They are used in three same sentences, that is the first, second, and fourth sentences; thus, the weight of the tie is 3.

These relational characteristics of each word in a document can reflect the unique characteristics of the document better than the frequency of each word. For example, according to the network information, we know that “banana” and “apple” are used together frequently; thus, it is likely that “banana” is used in the document in relation to “apple.” On the other hand, although “eggplant” is used frequently in the document, the weights of its ties with other words such as “apple” and “banana” are small. We know that “eggplant” might play a bridge role in the network; i.e., it is located on the path between other words, as shown in Fig 1.

In this study, for the relational characteristics of the words in a network, we focus on 1) centrality and 2) the number of times that two words are used in the same sentence in a document (i.e., the tie weight).

#### Centrality measures

Four types of centrality measures are frequently used in network analysis: degree, betweenness, closeness, and eigenvector centralities [19, 20]. These measures reflect the degree to which a node plays a central role in a network [21]. These centrality measures have also been used in studies about NLP tasks using network-based approaches [16, 22]. Centrality measures of words have been found to reflect the characteristics of a document [23]. We use these centrality measures of each word used in a document to vectorize the document.

1) Degree centrality. The degree centrality of a node in a network is defined as follows [19].

di=degreeimaximum_degreeg

where *degree*_*i*_ is the degree of node *i*, and *maximum*_*degree*_*g*_ is the maximum degree that a node can have in network *g*, which is *N*-1, where *N* is the number of nodes in *g*. The degree of a node refers to the number of nodes adjacent to that node. In a network of words (i.e., a document), the degree of a word refers to the number of other words used in the same sentence as the word. Thus, a word with a large degree centrality is used with more other words in the document than a word with a small degree centrality. In Fig 1, the size of a node is determined by the degree of the node, which is proportional to its degree centrality. The degree centrality of each word in DocA is presented in the first row of Table 2.

**Table 2:** Centralities of the words in the network of DocA.

Centrality	apple	banana	carrot	cherry	eggplant	grape	melon	orange	pear	spinach	watermelon
Degree	0.60	0.60	0.20	0.30	0.80	0.60	0.30	0.60	0.30	0.30	0.40
Betweenness	0.11	0.11	0.00	0.00	0.49	0.06	0.00	0.06	0.00	0.00	0.00
Closeness	0.71	0.71	0.45	0.53	0.83	0.71	0.53	0.71	0.53	0.56	0.53
Eigenvector	0.39	0.39	0.15	0.13	0.41	0.40	0.13	0.40	0.13	0.23	0.30

2) Betweenness centrality. The betweenness centrality of a node is defined as follows [19].

$$b_{i}=\sum_{s,t\in V}\frac{{\sigma (i)}_{s,t}}{\sigma_{s,t}}$$

where *V* is the set of nodes in the network, *σ*_*s*,*t*_ is the number of shortest paths between nodes *s* and *t*, and *σ*(*i*)_*s*,*t*_ is the number of shortest paths between nodes *s* and *t* that pass through node *i*. In this study, we considered unweighted ties when calculating betweenness centrality. The betweenness centrality indicates the degree to which a node plays a bridge role between other nodes in the network. The meaning of the betweenness centrality of a word in a word network can be vague. For illustration, consider a document consisting of the following two sentences.

Sentence 1: “The research shows that health is an important issue.”
Sentence 2: “The research also explains how media can play a critical role.”

**Fig 2:**
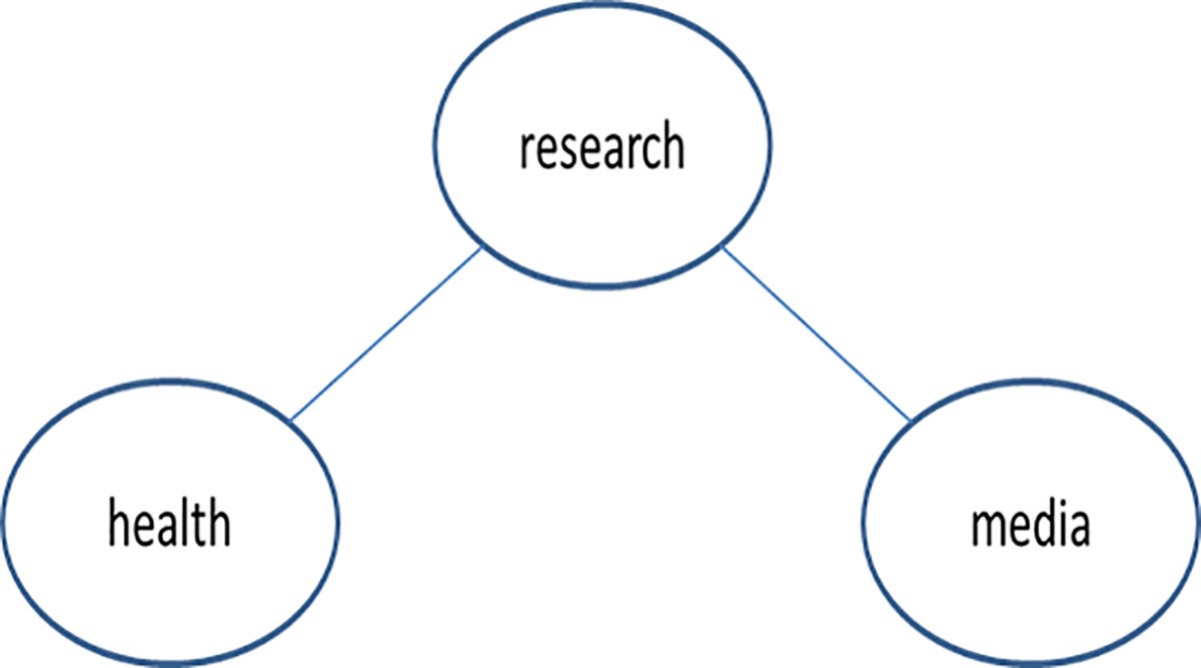
Example network.

A word network consisting of three words “research,” “health,” and “media” for the document is shown in Fig 2.

In the document represented in Fig 2, “research” plays a bridge role between “health” and “media.” That is, without “research,” “health” cannot be reached by “media,” and vice versa. The relationships between the three words can be interpreted in two ways. First, we can consider that the document discusses two specific aspects of “research”: “health” and “media.” Second, we can consider that the document discusses the same aspect of “health” and “media,” that is, “research” or discusses “health” and “media” from the “research” perspective.

The betweenness centralities of the words in DocA are presented in the second row of Table 2.

3) Closeness centrality. The closeness centrality of a node is defined as follows [19].

$$c_{i}=\frac{n-1}{\sum_{j=1}^{n-1}dis(i,j)}$$

where *n* is the total number of nodes in the network, and *dis*(*i*,*j*) is the distance between nodes *i* and *j*. In this study, we considered unweighted ties when calculating the distance between nodes. The closeness centrality of a node indicates how closely the node is connected to all the other nodes in the network. The closeness centrality of a word in a word network might indicate the degree to which the word is related to other words in the document, on average. A word with a large closeness centrality is more closely related to other words in the document, on average. That is, other words in the document likely possess some meaning that is related to the word having a large closeness centrality. For example, if the word “research” has a large closeness centrality in a document, other words in the network are likely to have some meaning related to “research.” The closeness centralities of the words in DocA are presented in the third row of Table 2.

4) Eigenvector centrality. The eigenvector centrality of a node is based on the principle that the centralities of other nodes to which the node is connected represent the centrality of the node [21]. The eigenvector centrality values of the nodes in a network are calculated using the eigenvectors of the adjacent matrix of the network, which is why the metric is called “eigenvector centrality.” In this study, we considered unweighted ties when calculating eigenvector centrality. The eigenvector centrality values of the words in DocA are presented in the last row of Table 2.

The aforementioned centrality values of words can be used to represent a document as a vector. For example, if we use the degree centrality values of words, for DocA, we have the vector (0.6, 0.6, 0.2, 0.3, 0.8, 0.6, 0.3, 0.6, 0.3, 0.3, 0.4). A vector of a document constructed using centrality values can be used for several purposes, such as document similarity and clustering analysis.

#### Number of times that a word is used with other words (tie weight)

In addition to the centrality measures, we consider the tie information between the words used in the document, especially the number of times that two words are used in the same sentence, which is known as the weight of the tie. The tie weight between words in a document can indicate the characteristics of the document, mainly because an author conveys his/her message in a document by connecting words, which can be captured by ties between words in the document. For DocA, we have 11 words, indicating that there are 55 possible pairs of two words. When there are many words, which is common in text analysis, it is impossible to use all the possible combinations of two words because of the large number of combinations. Instead, we select a certain number of words to construct a vector using their tie information. Several different methods can be used. First, we can select words according to their frequencies. Second, we can choose words according to our research questions or theoretical background. Consider the following five words from DocA: “apple,” “banana,” “eggplant,” “grape,” and “orange”; we have 10 pairs of two words. The value of each pair of two words is the number of times that those two words are used in the same sentence. For example, the two words “apple” and “banana” are used in the same sentence three times. The weight values for the 10 pairs of words are presented in Table 3.

**Table 3:** Weight values of the ties between two words of “apple,” “banana,” “eggplant,” “grape,” and “orange” in DocA.

Words pair	('apple', 'banana')	('apple', 'eggplant')	('apple', 'grape')	('apple', 'orange')	('banana', 'eggplant')	('banana', 'grape')	('banana', 'orange')	('eggplant', 'grape')	('eggplant', 'orange')	('grape', 'orange')
Tie weight	3	1	1	1	1	1	1	1	1	2

## Experiments

To evaluate how well the vectorization method using the network information about words represents the characteristics of a document, we compare the results of clustering analysis via vectors constructed using the relational characteristics of words (i.e., centralities and tie weights) with those obtained via TF and TFIDF-based vectors. For the clustering analysis, we use K-means and hierarchical clustering algorithms.

### Example document data

For the experiments, we use example text data composed of 14 documents pertaining to four different topics; thus, the number of clusters that we wish to find is four. The documents are news articles, each covering a particular topic. Three articles belong to the same cluster—Cluster 1—and discuss the release of a new battery case for the iPhone, which is known as a Smart Battery Case. The second cluster—Cluster 2—is composed of three other news articles concerning a shooting incident that occurred in Jacksonville, USA in 2018. Cluster 3 contains four news articles about basketball player Kevin Durant, in particular, the charity foundation that he established in Maryland, USA. The final cluster—Cluster 4—consists of four news articles about tennis player Serena Williams, specifically her loss in the 2019 Australian Open. Although articles in the same cluster discuss the same topic, the details of their contents differ. In the experiments, we test how well the 14 documents are clustered using the vectors developed via the new methods compared with the methods based on the TF and TFIDF.

#### Vectorization of the documents using words network information

To vectorize each of the fourteen documents, we used the noun words in the corpus, which were identified using the nltk module in Python. There were 869 noun words in the corpus. As explained in the preceding section, a tie between two noun words was defined to exist when they were used in the same sentence. When calculating centralities whose values are influenced by the number of nodes, which include degree, betweenness, and closeness centralities, we took into account the length of each document and the corpus. For this, we first divided each centrality measure by the number of noun words used in each document and multiplied it by the total number of noun words in the corpus. The eigenvector centrality is calculated using the eigenvector of the largest eigenvalue of the adjacency matrix of the network, thus, the centrality is not a function of the number of nodes [24]. To vectorize each document using the tie weight information, in this experiment, we only used top 10 percent words of the total words according to their frequencies in the corpus. This is mainly because the number of possible pairs of two words increases exponentially with the number of words. Further, because the weight of a tie between two words can vary with the length of the document, we divided tie weight by the number of words in the document.

#### Comparison using K-means clustering algorithm

For the 14 documents, a K-means algorithm was applied using the “scikit-learn” module in Python [25]. The K-means algorithm calculates the similarity between documents according to the Euclidean distance between their corresponding vectors [26]. With the hyperparameter for the number of clusters to be found set as 4, we ran several analyses using different vectors for the documents, that is, vectors of the 1) TF, 2) TFIDF, 3) centrality measures, and 4) tie weight information. The results of the clustering analyses using the different vectors are presented in Table 4.

**Table 4:** Results of the clustering analyses of the 14 documents.

Document ID	NMI*	0	1	2	3	4	5	6	7	8	9	10	11	12	13
Correct cluster ID	-	0	0	0	1	1	1	2	2	2	2	3	3	3	3
TF	1.0	0	0	0	1	1	1	2	2	2	2	3	3	3	3
TFIDF	1.0	0	0	0	1	1	1	2	2	2	2	3	3	3	3
Degree	1.0	0	0	0	1	1	1	2	2	2	2	3	3	3	3
Betweenness	0.70	0	0	0	2	1	3	0	0	0	0	1	1	1	1
Closeness	0.82	0	0	0	1	1	3	2	2	2	2	0	0	0	0
Eigenvector	1.0	0	0	0	1	1	1	2	2	2	2	3	3	3	3
Tie weight	0.66	1	0	0	1	1	1	2	3	2	2	1	1	1	1

* NMI: Normalized mutual information

Table 4 shows that the results of the clustering analyses based on vectors of the relational characteristics of the words are less accurate than the TF-based results, except for the analysis using the vectors of eigenvector centrality. In general, the similarity between documents based on the Euclidean distance does not work well for the vectors constructed using the relational characteristics of the words.

**Fig 3:**
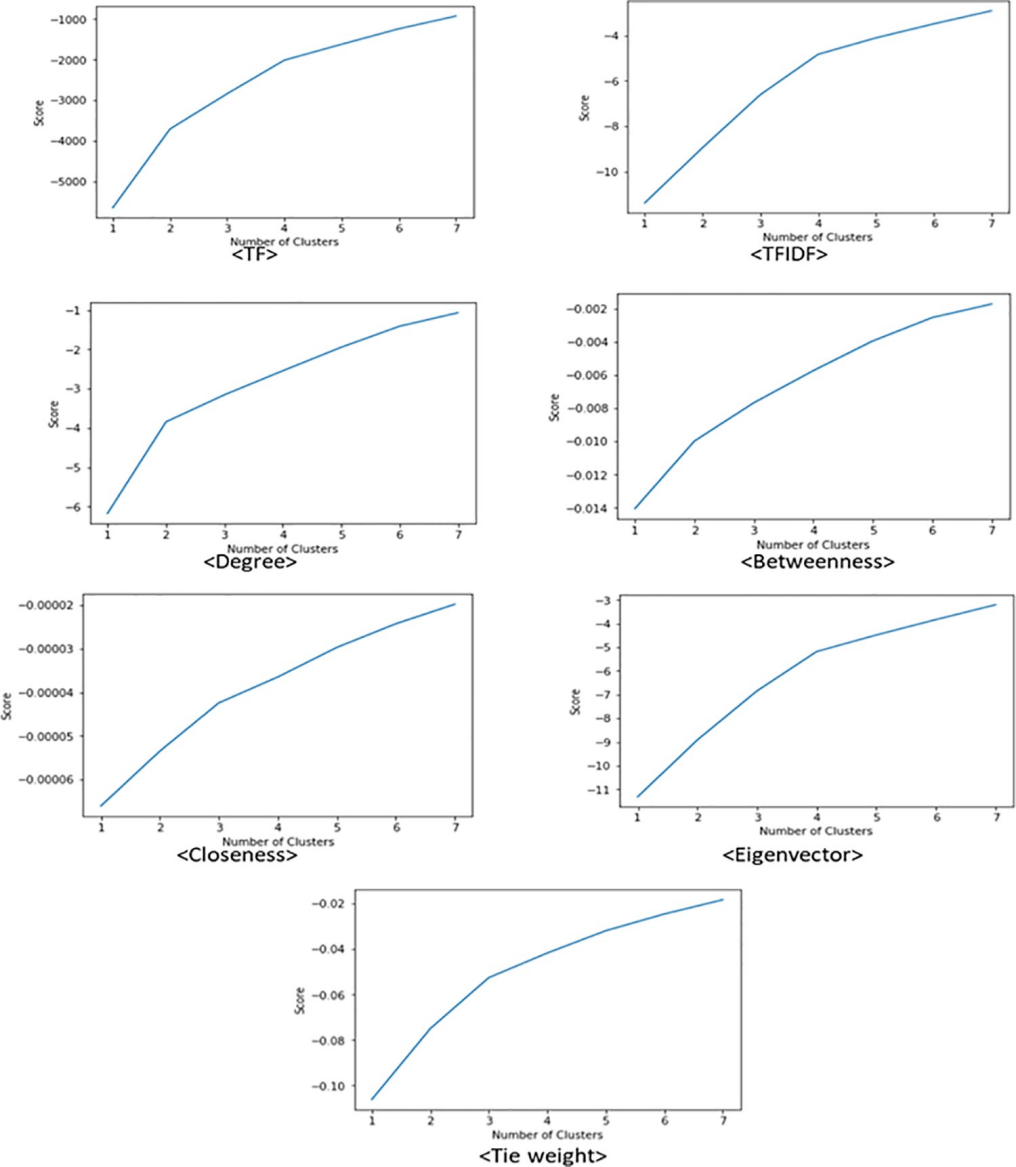
Score plots of each method. *Note*: Horizontal axis: Number of Clusters, Vertical axis: Score.

We also present plots of the score values for each analysis with different numbers of clusters, which indicate how accurately the analyses determine the number of clusters from the text data. The results are shown in Fig 3. The score value of K-means analysis with a particular number of clusters is calculated as follows [27].

$$score=-\sum_{i}{\left(d_{i,center}\right)}^{2}$$

where *d*_*i*, *center*_ is the distance between Doc *i* and the center of the cluster to which the document belongs.

If the clustering analysis returns the correct number of clusters, which is four in this case, the value of the score should increase rapidly up to the correct number of clusters and should increase slowly after the number. This is because dividing the corpus into more clusters than the optimal number of clusters leads to a small decrease in the sum of squared distances (i.e., the score value). The plots in Fig 3 indicate that the clustering analyses based on the vectors of the relational information about the words generated less accurate results than the analysis using the TF or TFIDF vectors, except for the case of the eigenvector centrality vectors. The plots for TF, TFIDF, and eigenvector centrality have a kink at the point where the number of clusters is 4.

#### Comparison using hierarchical clustering algorithm

To use the cosine similarity between vectors instead of the Euclidean distances, we employ a hierarchical clustering method, which is implemented in the “AgglomerativeClustering” class in Python [28]. For the “linkage” parameter, we use the “average” option, and for the “affinity” parameter, we used “cosine.” Similar to the K-means analysis, we set the number of clusters to be found as 4.

**Fig 4:**
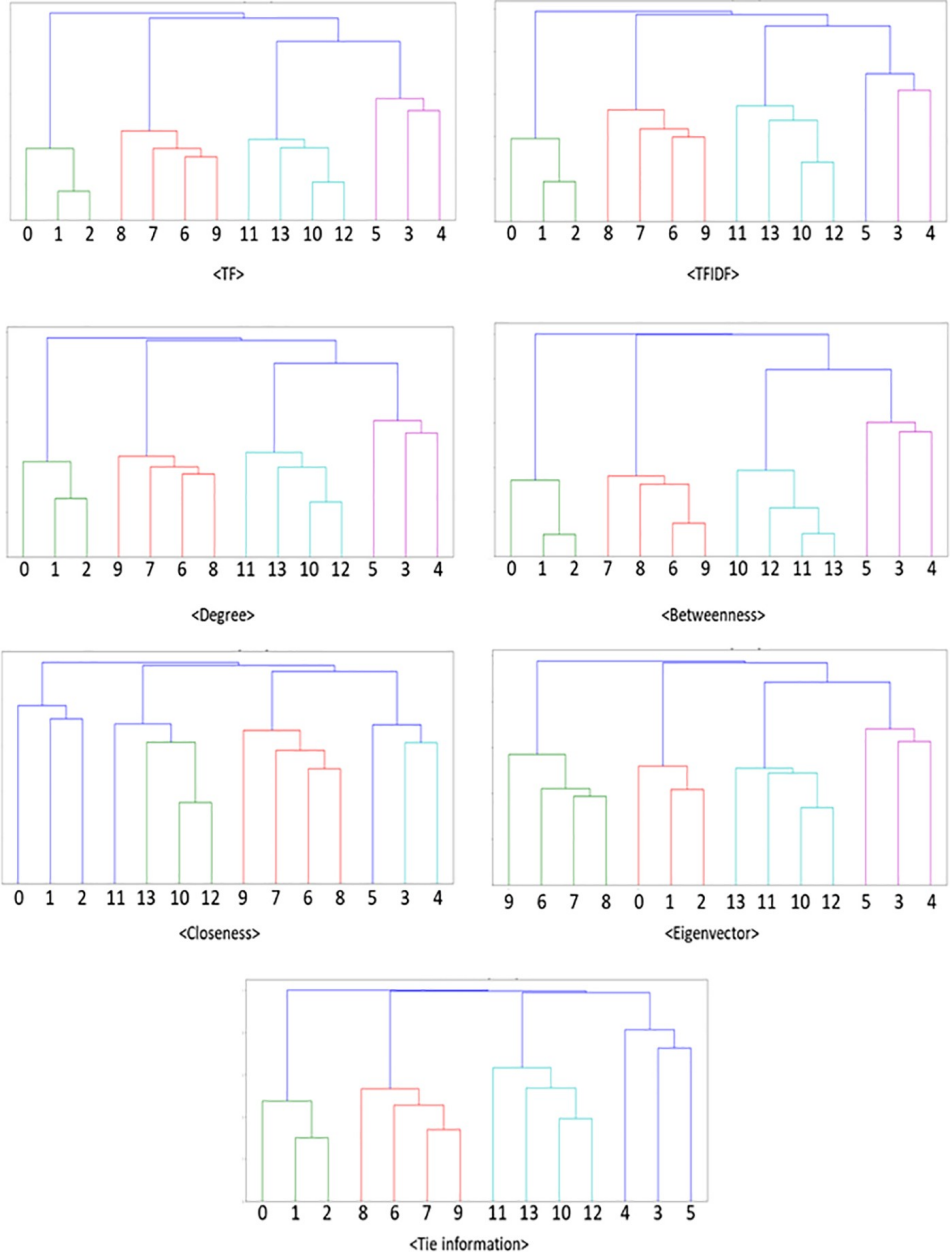
Dendrogram plots of the hierarchical clustering analyses. *Note*: Horizontal axis: Document ID, Vertical axis: Distance.

In contrast to the results of the K-means analyses, in the case of the hierarchical clustering analysis based on cosine similarities, all the methods based on different types of vectors returned the correct answer. That is, four clusters were correctly identified via all the vector methods. The dendrogram plots for each vector method are presented in Fig 4.

In a dendrogram, the values on the vertical line refer to the distance between documents or clusters. Thus, as the results of the clustering analysis are more correct, the distances between documents that belong to the same cluster should be small, while those between documents that belong to different clusters should be large. In addition to the dendrogram plots, to more accurately compare the performances of the different vector methods, we calculated the ratio of the similarity between documents in the same cluster to the similarities of the clusters.

Average similarity between documents in the same cluster, denoted as *A*_*T*_

To calculate *A*_*T*_, we first calculated the average consine similarity between documents in the same cluster, as follows:

avg_simC=∑i,j∈Ccosθ(doci,docj)(n2)

where *cosθ*(*doc*_*i*_,*doc*_*j*_) is the cosine similarity between documents *i* and *j* in cluster *C*, $\left(\begin{array}{c} n\\ 2 \end{array}\right)=\frac{n!}{2!(n-2)!}$, and *n* is the number of documents in cluster *C*.

Using *avg*_*sim*_*C*_ for each cluster, we obtained the following average similarity metric for all the clusters.

AT=∑C∈Tavg_simC#Clusters

Here, *T* indicates the total text data. In this case, there are four different clusters in the total data; thus, # *Clusters* = 4. A large *A*_*T*_ indicates that the similarity between the documents in the same cluster is high, on average.

Average similarity between clusters, denoted as *B*_*T*_

We also calculated the average similarity between clusters. For this, we first obtained the average vector for each cluster, as follows:

avg_vectorC=∑i∈Cvectori#vectorsinC.

Using the average_vector_*C*_ of each cluster, we calculated the average cosine similarity between the average vectors, as follows:

BT=∑p,q∈Tcosθ(avg_vectorp,avg_vectorq)#Clusters.

A small *B*_*T*_ indicates that the similarity between the clusters is low, on average.

The distinction between clusters is related to the ratio of *A*_*T*_ to *B*_*T*_, that is, $\frac{A_{T}}{B_{T}}$. If $\frac{A_{T}}{B_{T}}$ is large, it is likely that clusters are more accurately identified. The values of $\frac{A_{T}}{B_{T}}$ obtained via each vector method examined in this study are presented in Table 5.

**Table 5:** $\frac{A_{T}}{B_{T}}$ of each vector method.

	TF	TFIDF	Degree	Betweenness	Closeness	Eigenvector	Tie weight
*A*_T_*	0.614	0.514	0.555	0.639	0.342	0.473	0.472
*B*_T_**	0.070	0.047	0.074	0.035	0.100	0.068	0.006
ATBT	8.762	11.025	7.462	18.115	3.410	6.917	84.681

* AT=∑C∈Tavg_simC#Clusters

** BT=∑p,q∈Tcosθ(avg_vectorp,avg_vectorq)#Clusters

The results in Table 5 indicate that the value of $\frac{A_{T}}{B_{T}}$ obtained using vectors of the tie weights was the largest (84.681), followed by those obtained using betweenness centrality-based vectors (18.115) and TFIDF-based vectors (11.025). Thus, vectors constructed using tie weights between words in a document can more accurately identify clusters composed of distinctive documents than vectors constructed using other methods, especially the traditional methods (TF and TFIDF).

## Conclusion

We proposed a new method for vectorizing a document according to the relational characteristics of the words in the document. For the relational characteristics, we used four centrality measures and the tie weights between words. We suggested these methods because in some cases, information about the relations of a word to other words in a document can better represent the unique characteristics of the document than frequency-based methods, e.g., the TF and TFIDF. Thus, machine learning algorithms (e.g., clustering algorithms) based on the similarity between documents can generate more accurate results by using vectors of the relational characteristics of words than by using vectors of the TF or TFIDF. In general, the results of clustering analysis based on the Euclidean distances between document vectors obtained using the proposed methods were inaccurate compared with the results obtained using vectors of traditional methods. However, the results obtained using cosine similarities between vectors of the new methods were comparable to those obtained using the vectors of traditional methods. In particular, the clustering analysis using vectors comprising the tie weights between words generated the most remarkable result. One limitation of this study is that we used a small number of documents for the clustering analysis. Although the results obtained from the small example dataset can hardly be generalized, they suggest that at least in some cases, vectorization of a document using the relational characteristics of the words in the document can provide more accurate results than traditional methods.

## Supporting information

S1 Dataset (ZIP)
